# Dependence of Proximal GC Boxes and Binding Transcription Factors in the Regulation of Basal and Valproic Acid-Induced Expression of t-PA

**DOI:** 10.1155/2016/7928681

**Published:** 2016-02-07

**Authors:** Erik Ulfhammer, Pia Larsson, Mia Magnusson, Lena Karlsson, Niklas Bergh, Sverker Jern

**Affiliations:** The Wallenberg Laboratory for Cardiovascular Research, Institute of Medicine, Sahlgrenska Academy, University of Gothenburg, 413 45 Gothenburg, Sweden

## Abstract

*Objective.* Endothelial tissue-type plasminogen activator (t-PA) release is a pivotal response to protect the circulation from occluding thrombosis. We have shown that the t-PA gene is epigenetically regulated and greatly induced by the histone deacetylase (HDAC) inhibitor valproic acid (VPA). We now investigated involvement of known t-PA promoter regulatory elements and evaluated dependence of potential interacting transcription factors/cofactors.* Methods.* A reporter vector with an insert, separately mutated at either the t-PA promoter CRE or GC box II or GC box III elements, was transfected into HT-1080 and HUVECs and challenged with VPA. HUVECs were targeted with siRNA against histone acetyl transferases (HAT) and selected transcription factors from the Sp/KLF family.* Results.* An intact VPA-response was observed with CRE mutated constructs, whereas mutation of GC boxes II and III reduced the magnitude of the induction by 54 and 79% in HT-1080 and 49 and 50% in HUVECs, respectively. An attenuated induction of t-PA mRNA was observed after Sp2, Sp4, and KLF5 depletion. KLF2 and p300 (HAT) were identified as positive regulators of basal t-PA expression and Sp4 and KLF9 as repressors.* Conclusion.* VPA-induced t-PA expression is dependent on the proximal GC boxes in the t-PA promoter and may involve interactions with Sp2, Sp4, and KLF5.

## 1. Introduction

The majority of myocardial infarctions and ischemic strokes are caused by intravascular clotting that, when unopposed, rapidly can progress into an occluding arterial thrombus. To protect against this, the vascular endothelium of healthy individuals has the ability to acutely release the fibrinolytic enzyme tissue-type plasminogen activator (t-PA) [1], causing the clot to dissolve. In individuals with impaired capacity for t-PA release, the coagulation/fibrinolytic balance may be shifted in favor of thrombosis and consequently intravascular thrombus formation may propagate and lead to tissue infarction. Interestingly, t-PA release has been found to be defective in conditions associated with increased risk of thrombotic events, such as hypertension [2, 3], obesity [4], and coronary atherosclerosis [5, 6].

Consequently, there is a clinical need to find a pharmacological tool for prevention of atherothrombotic events that targets the endogenous fibrinolytic system. We recently showed that valproic acid (valproate, 2-propylpentanoic acid, VPA), a clinically available antiepileptic and mood-stabilizing drug, powerfully upregulated t-PA in cultured endothelial cells and enhanced t-PA release in atherosclerotic man as well as in a porcine coronary ischemia model [7–9]. This effect was observed at clinically relevant concentrations and is likely due to the histone deacetylase (HDAC) inhibitory effect of VPA [10, 11]. Similarly, t-PA expression was strongly induced by classical (butyrate, trichostatin A) as well as other newer histone deacetylase inhibitors (HDACi) [12–15].

However, the mechanism by which VPA induces t-PA expression is currently unknown. Based on our previous findings, we hypothesize that VPA mediates its effect by inhibiting HDACs. This causes a more acetylated histone status around the t-PA promoter, resulting in a permissive and transcriptionally active chromatin state. When the t-PA promoter is accessible, the binding or posttranslational modifications of transcription factors can mediate induction via cis-acting elements. If this hypothesis is correct, dysfunctionality of critical cis-element(s) or unavailability of key transcription factors would have great impact on the responsiveness to VPA.

The t-PA gene is transcribed primarily from a TATA-independent transcription initiation site (TIS) [18, 19]. Three elements in the t-PA promoter, just upstream of the TIS, have previously been reported to be important for t-PA expression: one cyclic adenosine monophosphate (cAMP) responsive element- (CRE-) like site and two GC boxes (II and III) [16, 17]. Using a transfection approach, we examined the importance of these elements during VPA stimulation. The role of relevant transcription factors interacting with these elements was investigated in siRNA experiments.

## 2. Material and Methods

### 2.1. Ethics Statement

The investigation conformed to the principles of the Declaration of Helsinki. Use of human umbilical cords cells was approved by the Regional Ethics Review Board of Gothenburg (number 449-93). The personnel at the maternity ward of the Sahlgrenska University Hospital were given verbal informed consent from the donors regarding the use of umbilical cord cells for research purposes. Given that the study material was nonidentifiable, written consent was uncalled for and the use of oral consent was approved by the ethical review board.

### 2.2. Chemicals

Na valproate was purchased from Sigma-Aldrich (St. Louis, MO, USA) and stock solutions (0.3 M) were prepared in complete endothelial cell culture medium (EGM-2, Lonza, Basel, Switzerland) and stored at −80°C. The compound was diluted in cell culture medium immediately before use.

### 2.3. Cell Culture

Due to their suitability as model system for transfection of large reporter constructs, HT-1080 fibrosarcoma cells were utilized in reporter studies. HT-1080 cells (kind gift from Professor Robert Medcalf, Monash University, Australia) were cultured in DMEM (Invitrogen) supplemented with 10% fetal bovine serum and penicillin/streptomycin. The validity of using HT-1080 was ensured by verifying a consistent and significant induction of t-PA mRNA upon stimulation with 3 mM VPA.

Human umbilical vein endothelial cells (HUVECs) were used as a physiologic relevant cell type in both siRNA studies and confirming reporter studies. HUVECs were extracted by collagenase (Sigma-Aldrich) treatment [20] of fresh umbilical cords obtained from the maternity ward of the Sahlgrenska University Hospital. Cells were cultured in EGM-2 medium and all experiments were performed in passage 1. All reporter and siRNA experiments were performed in triplicate and duplicate, respectively. HUVEC experiments were repeated on cells from a minimum of three different donors while HT-1080 experiments were repeated on three different occasions.

### 2.4. Reporter Constructs and Site-Directed Mutagenesis

**Figure 1:**
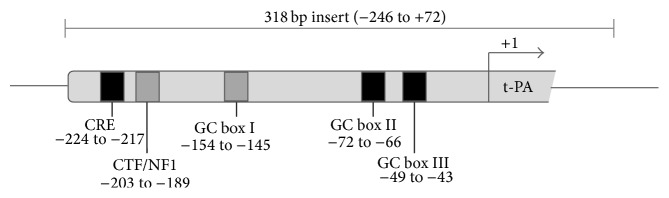
Schematic illustration of the proximal promoter region of the t-PA gene and the coverage of the insert used in presented reporter studies. Marked boxes illustrate previously described cis-acting elements [16, 17]; the cAMP responsive element- (CRE-) like site, the CAAT-binding transcription factor/nuclear factor-1- (CTF/NF1-) like binding site, and the three GC box elements (I, II, and III). Highlighted in black are elements that were subjected to site-directed mutagenesis and tested for involvement in induction by VPA. All positions in this study are given in relation to the major t-PA transcription initiation site (TIS, +1, positioned on chromosome 8:42,207,565) [18] and are according to the Dec. 2013 Genome Reference Consortium GRCh38 assembly obtained from the UCSC Genome Browser database (http://genome.ucsc.edu/).

318 bp of the proximal t-PA promoter (−246 to +72) was PCR amplified from the insert of the previously described t-PA9578-CAT vector [21] and cloned into the XhoI/HindIII sites of a firefly luciferase reporter vector (pGL4-Luc, Promega, Madison, WI, USA). Subsequently, the construct was verified by sequencing on a 3730 DNA analyzer (Applied Biosystems, Foster City, CA, USA). Site-directed mutagenesis of the t-PA CRE site and the GC box II and GC box III elements was performed by GenScript Corporation (Piscataway, NJ, USA). A schematic view of the proximal promoter region of the t-PA gene is given in Figure 1. To create t-PA-318-CREmut-pGL4, the t-PA CRE site TGACATCA was mutated to **G**G**CT**ATCA, to create t-PA-318-GCIImut-pGL4, the GC box II site CCCGCCC was mutated to C**A**CGC**A**C, and to create the t-PA-318-GCIIImut-pGL4, the GC box III site CCCACCC was mutated to **A**C**T**A**GT**C. These particular base pair substitutions have previously been reported to interrupt transcription factor binding to these sites [22, 23]. All mutated constructs were verified by sequencing.

### 2.5. Transient Transfection of Reporter Constructs

On the day prior to transfection, cells were seeded at a density of 100 000 cells/well in 12-well plates (HT-1080) or 50 000 cells/well in 24-well plates (HUVECs). Cells were transfected using the FuGENE 6 (HT-1080) or FuGENE HD (HUVECs) reagent according to manufacturer's protocol (Promega) using a mixture of 500 ng of t-PA promoter firefly luciferase construct and 10 ng of the SV40 driven pGL4.73 renilla luciferase control vector. On the following day, cells were treated with 3 mM of VPA for 24 h after which cells were harvested in passive lysis buffer (Promega) and analyzed on a GloMax® Luminometer using Dual Luciferase Assay reagents (Promega). Firefly luciferase signals were normalized to renilla luciferase signals and data are presented relative to unstimulated controls.

### 2.6. Short Interfering RNA Transfection

Short interfering RNAs (siRNAs) specific for selected Sp/KLF transcription factors, p300, and PCAF were obtained from Dharmacon (Thermo Fisher Scientific, Lafayette, CO, USA). The selection criteria for Sp/KLF factors were either a significant regulation (*p* < 0.001) by VPA in a microarray experiment [7] or previously having been described in an endothelial gene regulation context. Each specific ON-TARGETplus SMART pool siRNA set is listed in Online Resource 1 in Supplementary Material available online at http://dx.doi.org/10.1155/2016/7928681. A detailed siRNA protocol has been presented elsewhere [7]. Briefly, HUVECs were transfected twice (0 and 24 h) using DharmaFECT 4 transfection reagent (Dharmacon), fresh medium was added at 48 h, and cells were stimulated with 3 mM VPA at 72 h, and at 96 h cells were harvested for mRNA analysis of target reduction and t-PA gene expression. Each siRNA was used in a final concentration of 10 nM, except Sp2 and KLF6 (20 and 40 nM, resp.). Results were only used when target reduction was at least 75%. Two negative controls were used for siRNA; in one the DharmaFECT 4 transfection reagent was added alone to cells (mock); in another a control siRNA was used (All Star Negative control, Qiagen). No difference in expression of t-PA or target gene was observed with either control.

### 2.7. Real-Time PCR

Total RNA was prepared using RNeasy Mini RNA kit (Qiagen, Hilden, Germany) and genomic DNA was removed using RNase-free DNase I set (Qiagen). Levels of t-PA, Sp1, Sp2, Sp3, Sp4, KLF2, KLF4, KLF5, KLF6, KLF9, KLF10, KLF13, KLF15, p300, and PCAF mRNA were analyzed with real-time PCR, performed on an Applied Biosystems 7500 Fast Real-Time PCR System using cDNA and Taqman reagents obtained from Applied Biosystems. t-PA mRNA was analyzed with a Custom Taqman Expression Assay (Applied Biosystems) using the following sequences: t-PA forward primer: 5′-GGC CTT GTC TCC TTT CTA TTC G-3′, t-PA reverse primer: 5′-AGC GGC TGG ATG GGT ACA G-3′, and t-PA probe 5′-TGA CAT GAG CCT TCA GCC GCT-3′. Confirmation of knock-down of respective Sp/KLF factor and PCAF was performed using Taqman Gene Expression Assays (Applied Biosystems) and of p300 using a QuantiTect Primer Assay from Qiagen (all listed in Online Resource 1). HPRT was used as endogenous internal standard in all experiments except in KLF9 studies where GUSB was used.

### 2.8. Statistics

Data are presented as mean and standard error of the mean (SEM). The statistical evaluation was performed using paired Student's *t*-test and a *p* value of less than 0.05 was considered statistically significant. All comparisons are specified in the figure legends and were performed between samples from the same experiment and timepoint. The* primary* focus of this study was to examine if the VPA-response could be modulated by an approach involving site-directed mutagenesis of regulatory t-PA promoter elements or by siRNA mediated knockdown of candidate transcription factors interacting with the same. The* secondary* focus of the study was to evaluate if siRNA knock-down of the transcription factors affects basal t-PA gene expression. For the latter, Bonferroni correction for multiple analysis was performed.

## 3. Results

### 3.1. Mutation of GC Boxes Attenuates VPA-Induced t-PA Promoter Activity

**Figure 2:**
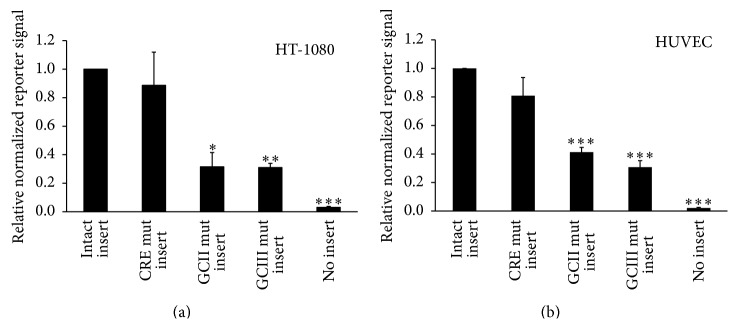
Basal signal levels of t-PA reporter construct and its dependence on proximal GC boxes. HT-1080 (a) and HUVEC (b) cells were cotransfected with a firefly luciferase construct containing different variants of a 318 bp insert of the t-PA proximal promoter and a SV40 driven pGL4.73 renilla luciferase control vector. Firefly luciferase signals were normalized to renilla luciferase. The figures show relative basal signal levels in unstimulated reporter constructs and mean values of three (HT-1080) and four (HUVEC) independent experiments. The variants of the t-PA insert were unmutated (intact), CRE mutated, GC box II mutated, GC box III mutated, and no insert (empty pGL4.10 vector). Statistical evaluation was performed using paired Student's *t*-test and is made relative to constructs with intact insert. ^*∗*^ *p* < 0.05, ^*∗∗*^ *p* < 0.01, and ^*∗∗∗*^ *p* < 0.001.

**Figure 3:**
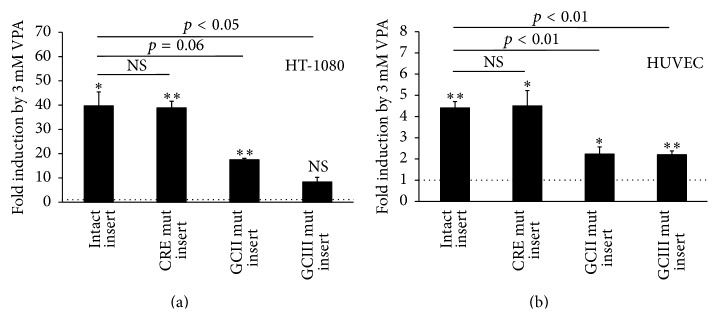
Induction of t-PA reporter construct by VPA and its dependence on proximal GC boxes. HT-1080 (a) and HUVEC (b) cells were cotransfected with a firefly luciferase construct containing different variants of a 318 bp insert of the t-PA proximal promoter and a SV40 driven pGL4.73 renilla luciferase control vector. Firefly luciferase signals were normalized to renilla luciferase and the figures show relative data and mean values of three (HT-1080) and four (HUVEC) independent experiments. The variants of the t-PA insert were unmutated (intact), CRE mutated, GC box II mutated, and GC box III mutated. After overnight incubation, cells were stimulated with 3 mM VPA for 24 h. Data are presented as relative inductions of respective construct upon stimulation with VPA, where the dotted line in each figure represents untreated controls (=1). Statistical evaluation was performed using paired Student's *t*-test and comparisons are relative to respective untreated control. The marks indicate the statistical comparison of the VPA-induction in the t-PA construct with intact insert* versus *the inductions of respective mutated insert. ^*∗*^ *p*<0.05; ^*∗∗*^ *p* < 0.01 and NS = nonsignificant.

To evaluate the role of the t-PA CRE and GC box II and III elements in basal and VPA-induced t-PA promoter activity, HT-1080 cells were transfected with nonmutated and mutated variants of a 318 bp insert of the t-PA proximal promoter (Figures 2(a) and 3(a)). Nonmutated constructs showed an approximately 40-fold induction (*p* < 0.05) with 3 mM VPA (Figure 3(a)). Mutation experiments showed no major involvement of the t-PA CRE element, as mutation of this site was unable to affect neither basal (Figure 2(a)) nor VPA challenged signal levels (Figure 3(a)). However, the importance of GC boxes II and III was evident as a 70% fall in basal signal was observed after mutation of either site (*p* < 0.05 and *p* < 0.01, resp.). Moreover, VPA-mediated induction of either construct was greatly attenuated by each mutation. Mutation of GC boxes II and III lowered the induction from being 40-fold in unmutated constructs to being 17- (*p* = 0.06) and 8-fold (*p* < 0.05), respectively. The basal signal level in empty pGL4.10 vector (no t-PA insert) was 3% of the level in constructs with intact insert.

To validate the responses observed in HT-1080 cells, similar reporter experiments were conducted in the more physiologic relevant cell type HUVEC. These cells showed lower transfection efficiency, reflected by overall lower signal levels and a VPA-induction of the intact insert that was only 10% of the corresponding induction in HT-1080 cells. Despite this, the effect of each individual mutation was almost identical between both cell types. No effect of the CRE-mutation was observed in HUVECs, while mutation of GC boxes II and III resulted in 59 and 69% lower basal signal levels (both *p* < 0.001) (Figure 2(b)). Similarly, VPA-induction was depressed from being 4.4-fold in unmutated constructs to being 2.2-fold in both GC box II and III mutated constructs (both *p* < 0.01) (Figure 3(b)).

### 3.2. siRNA Knock-Down of Sp2, Sp4, and KLF5 Attenuates t-PA mRNA Induction by VPA

To identify candidate transcription factors binding the two promoter GC boxes and to evaluate their involvement in the VPA-response, a set of Sp and KLF factors were individually knocked down with siRNA. Each tested siRNA resulted in a target reduction of 75% or below at 96 h, except KLF10 which was only depleted by 68%. Since we failed to deplete KLF10 mRNA to cut-off levels, t-PA mRNA was not analyzed in these samples.

**Figure 4:**
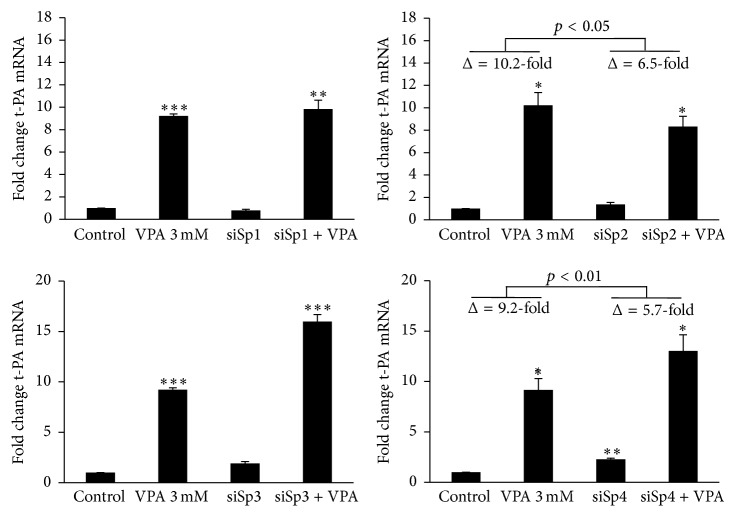
t-PA mRNA expression after siRNA mediated knock-down of Sp factors. HUVECs were treated with siRNA targeting Sp1, Sp2, Sp3, or Sp4 for 72 h and then stimulated with 3 mM VPA for 24 h. Statistical comparisons are made relative to untreated control cells. The marks indicate the statistical comparisons of the VPA-induction in nontransfected cells* versus* the VPA-induction in transfected cells (Δ = change in t-PA mRNA expression after VPA stimulation). ^*∗*^ *p* < 0.05, ^*∗∗*^ *p* < 0.01, and ^*∗∗∗*^ *p* < 0.001. The results are presented as mean values ± SEM of four independent experiments. The statistical evaluation was performed using paired Student's *t*-test together with Bonferroni correction for multiple analysis. Not shown in the illustrations are transfection controls with transfection reagent only (mock) and transfection with “All Star Negative” control siRNA (ASN) that were included in each experiment. These negative controls showed no significant effect on either t-PA or target mRNA.

**Figure 5:**
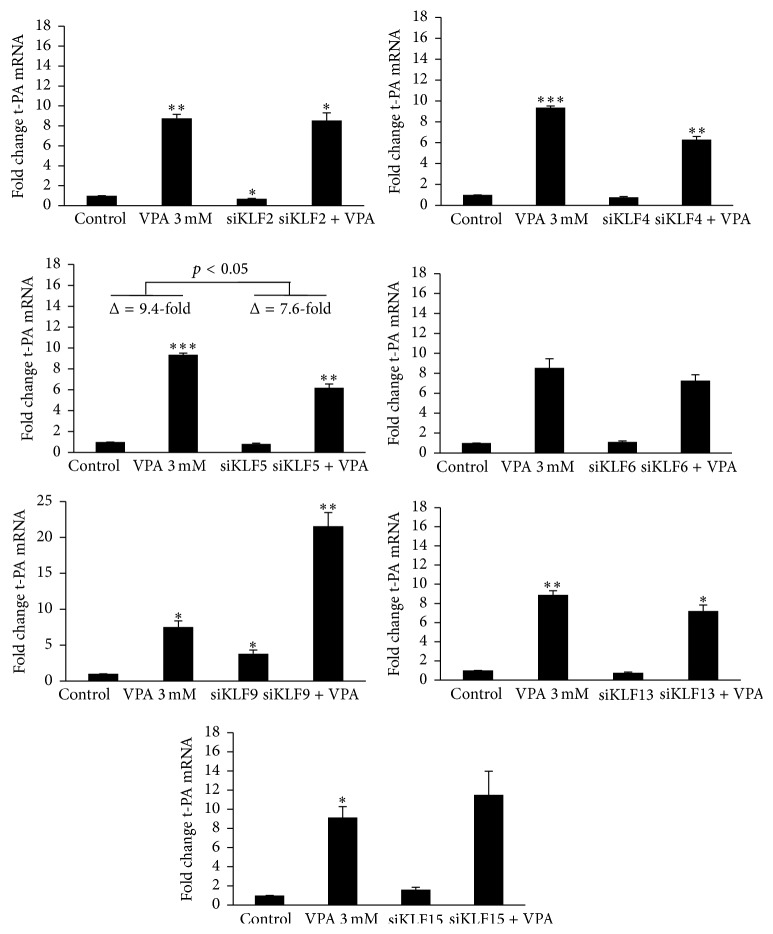
t-PA mRNA expression after siRNA mediated knock-down of KLF factors. HUVECs were treated with siRNA targeting KLF2, KLF4, KLF5, KLF6, KLF9, KLF13, or KLF15 for 72 h and then stimulated with 3 mM VPA for 24 h. Statistical comparisons are made relative to untreated control cells. The mark indicates the statistical comparison of the VPA-induction in nontransfected cells* versus* the VPA-induction in transfected cells (Δ = change in t-PA mRNA expression after VPA stimulation). ^*∗*^ *p* < 0.05, ^*∗∗*^ *p* < 0.01, and ^*∗∗∗*^ *p* < 0.001. The results are presented as mean values ± SEM of three (KLF6), four (KLF2, KLF4, KLF5, KLF13, and KLF15), or five (KLF9) independent experiments. The statistical evaluation was performed using paired Student's *t*-test together with Bonferroni correction for multiple analysis. Not shown in the illustrations are transfection controls with transfection reagent only (mock) and transfection with “All Star Negative” control siRNA (ASN) that were included in each experiment. These negative controls showed no significant effect on either t-PA or target mRNA.

Confirming our recent data [7], an approximately 10-fold induction of t-PA mRNA was observed with 3 mM VPA for 24 h. Single knock-down of each of the selected Sp and KLF transcription factors did not completely counteract the VPA-response (Figures 4 and 5). However, the magnitude of the t-PA mRNA induction was clearly diminished in Sp2 and Sp4 depleted cells (Figure 4) and modestly lowered in KLF5 knock-down cells (Figure 5). The absolute numbers of the VPA-induction in nontransfected cells compared to Sp2, Sp4, and KLF5 knocked cells changed from 10.2- to 6.5-fold (*p* < 0.05), from 9.2- to 5.7-fold (*p* < 0.01), and from 9.4- to 7.6-fold (*p* < 0.05), respectively.

**Figure 6:**
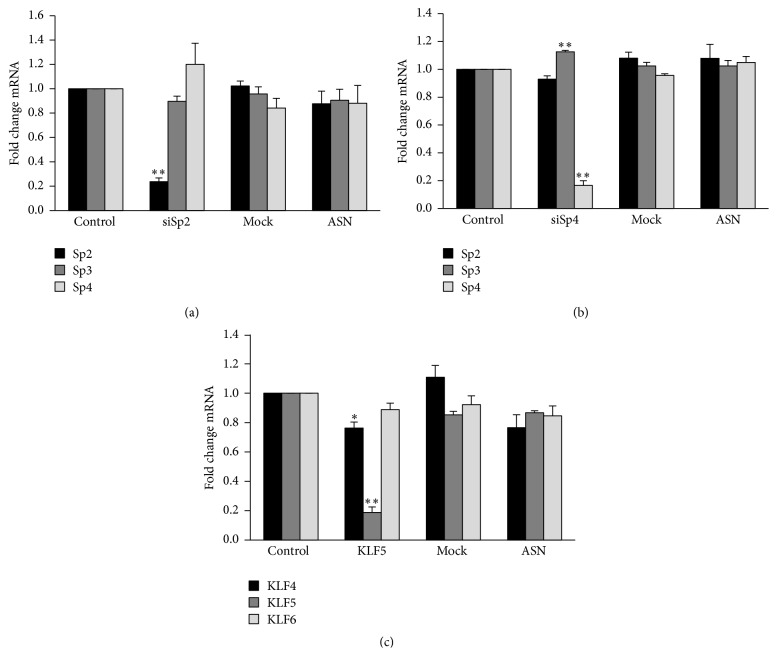
siRNA specificity in Sp2, Sp4, and KLF5 knocked HUVECs. mRNA expression of Sp2, Sp3, and Sp4 in HUVECs treated with siRNA targeting (a) Sp2 and (b) Sp4. (c) mRNA expression of KLF4, KLF5, and KLF6 in HUVECs treated with siRNA targeting KLF5. Mock = transfection reagent only; ASN = transfection with “All Star Negative” control siRNA. The results are presented as mean values ± SEM of three independent experiments. The statistical evaluation was performed using paired Student's *t*-test.

To verify the specificity of these positive hits on the VPA-response, each of the three siRNAs was tested for potential off-target effects on two other related mRNAs (Figure 6). All siRNA showed target reduction below 75% but a maximum effect of approximately ±20% on each of the other tested mRNAs.

### 3.3. siRNA Knock-Down of Sp4, KLF2, and KLF9 Affects Basal t-PA mRNA Expression

Evaluation of basal t-PA mRNA expression after siRNA knock-down of each factor revealed enhanced expression with depletion of Sp4 and KLF9 (Figures 4 and 5). Respective elevation of t-PA gene expression was 2.3- (*p* < 0.01) and 3.8- (*p* < 0.05) fold relative to nontransfected cells. Moreover, a significant fall in t-PA expression was observed with knock-down of KLF2 (−31%, *p* < 0.05).

The induction of basal t-PA expression in response to knock-down of either Sp4 or KLF9 was reflected by an elevated magnitude of the t-PA response after VPA stimulation. Relative to nontransfected control cells, Sp4 and KLF9 knocked cells showed VPA-inductions in the order of 13- and 22-fold, respectively.

### 3.4. Knock-Down of p300 Suppresses Basal t-PA mRNA Expression While Neither p300 Nor PCAF Depletion Affects Its Induction by VPA

**Figure 7:**
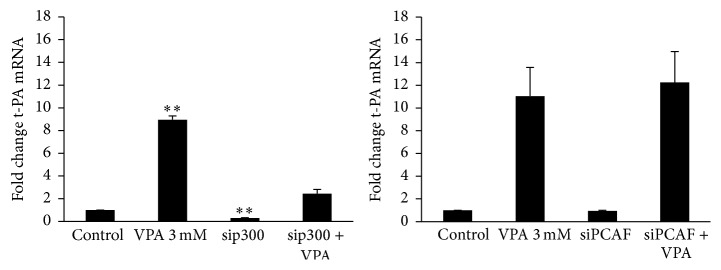
t-PA mRNA expression after siRNA mediated knock-down of p300 and PCAF. HUVECs were treated with p300 or PCAF-targeting siRNAs for 72 h and then stimulated with 3 mM VPA for 24 h. Statistical comparisons are made relative to untreated control cells. ^*∗∗*^ *p* < 0.01. The results are presented as mean values ± SEM of three independent experiments. The statistical evaluation was performed using paired Student's *t*-test together with Bonferroni correction for multiple analysis. Not shown in the illustrations are transfection controls with transfection reagent only (mock) and transfection with “All Star Negative” control siRNA (ASN) that were included in each experiment. These negative controls showed no significant effect on either t-PA or target mRNA.

A role of the histone acetyl transferases (HATs) p300 and PCAF has recently been proposed in t-PA induction by the HDACi MS-275 [13]. Therefore, we tested siRNA against these HATs. Depletion of p300 suppressed basal t-PA mRNA expression by 69% (*p* < 0.01) (Figure 7). VPA-mediated induction was reduced from 9- to 2.5-fold relative to untreated control cells. However, the induction of t-PA in transfected versus nontransfected control cells was almost identical (9- compared to 8-fold, *p* = NS). Treatment of cells with PCAF siRNA had no effect on either basal or VPA-induced t-PA mRNA expression.

## 4. Discussion

Today there is no clinically available pharmacological tool to enhance or restore an impaired endogenous fibrinolysis. Therefore, the potent induction of t-PA by the clinically used antiepileptic drug valproic acid (VPA) opens up an opportunity for pharmacological modulation of the system. Thus, it is important to clarify the exact mechanism by which VPA induces t-PA.

In our recent paper on VPA, we showed that the induction of t-PA is most likely due to the HDAC inhibitory effect of the drug, as we observed significant increases of acetylated histones H3 and H4 associated with the t-PA promoter after VPA-treatment [7]. In support of this, we observed no induction of t-PA with the VPA analogue valpromide (VPM), a substance that lacks HDACi activity [11, 24]. Further, no individual HDAC enzyme from subfamily I, IIa, or IV was critical for the induction by VPA, although an attenuated VPA-response was observed with single knock-down of HDAC3, HDAC5, and HDAC7. As VPA is a pan-HDAC enzyme inhibitor, we hypothesize that VPA mediates its effect on t-PA by acting on several different HDAC enzymes, thereby changing the acetylation status of histones surrounding the t-PA promoter. Consequently, this favors a more “receptive” and transcriptionally active chromatin state, thus opening up for the transcription machinery to access functional cis-regulatory elements. The aim of this study was to further investigate the VPA-response by exploring the importance of central cis-elements in the proximal t-PA promoter and known/stipulated transcription factors binding these elements.

Transcription of the human t-PA gene is predominantly initiated from a TATA-less transcription initiation site [18, 19] and the promoter region contains several well-characterized cis-acting elements. Of these, a CRE-like element (bp −224 to −217) and two GC boxes (GC boxes II and III, bp −72 to −66 and bp −49 to −43, resp.) are important for both constitutive and inducible activation of the t-PA promoter [16, 17, 19, 22]. Our data indicate no major involvement of the CRE site for either basal or VPA-stimulated t-PA promoter activity, although we observed a slight fall in signal levels with CRE mutated constructs. However, our data confirm the central importance of GC boxes II and III [16, 17], as we observed a striking fall in basal signal levels with mutagenesis of respective site. Interestingly, we also observed diminished VPA-responses with both mutations, indicating that these two sites are critical also for the induction by VPA. In light of these findings and the ability of transcription factors from the Sp/KLF family to bind GC/GT boxes [25], we then focused on identifying candidate Sp and KLF factors binding the t-PA GC boxes during basal and VPA-stimulated conditions.

With respect to the common view of dependence on Sp1 for t-PA expression, it was somewhat surprising that the Sp1 knock-down experiments showed unaltered t-PA expression during both control and VPA-treated conditions. One explanation could be an insufficient depletion of Sp1 protein. To dismiss this doubt, we confirmed knock-down of Sp1 protein using Western blotting and validated mRNA suppression of the positive control eNOS [26] in Sp1 depleted samples (data not shown). However, one should bear in mind that previous observations mainly are based on* in vitro* approaches; no one has actually confirmed a correlation between Sp1 and altered t-PA expression in an intact chromatin context.

In contrast to Sp1, our data indicate that Sp2 and Sp4 are essential for regulation of t-PA expression. The t-PA gene was induced upon knock-down of Sp4, thus suggesting that this factor acts as a repressor of the t-PA gene under constitutive conditions. Moreover, VPA-induction of t-PA mRNA was attenuated in Sp2 and Sp4 depleted samples. If the VPA-response was independent of Sp4, one would expect a relatively stronger t-PA induction after depletion of this repressive factor. Instead, it is possible that VPA mediates acetylation of the Sp4 protein itself, since HDAC inhibitors are known to acetylate many nonhistone proteins including transcription factors [27]. This may change Sp4 into an activator of the t-PA gene and/or favor enhanced binding to the t-PA promoter. The former mechanism has previously been reported for Sp3 [28]. The attenuated VPA-responses in Sp2 and KLF5 depleted cells could also be assigned to an acetylating effect, but the lack of involvement in basal t-PA expression indicates that the induction by VPA to a larger extent relies on recruitment of these transcription factors.

Although an intact VPA-response was observed after depletion of each of the other tested KLF factors, KLF2 and above all KLF9 showed involvement in basal t-PA gene expression. Our data indicate that KLF2 acts as an activator of t-PA expression. Interestingly, this feature of KLF2 is in line with extensive documentation on antithrombotic and anti-inflammatory properties of this factor (reviewed in [29]). Regarding KLF9, the literature is scarce and, to our knowledge, it has never been described in the context of t-PA regulation. Despite lack of involvement in the VPA-response, the profound induction of t-PA upon knock-down indicates a repressor function of KLF9. Interestingly, this is in line with existing literature [30] and certainly encourages future extending studies on the role of KLF9 in regulation of t-PA expression.

Dunoyer-Geindre and Kruithof recently evaluated the involvement of the HATs p300/CBP and PCAF in t-PA induction after using the HDACi MS-275. Based on the documented selectivity of the pharmacologic inhibitors used in the study, they proposed a role for PCAF rather than p300/CBP [13]. In order to test if these results are valid for the induction by VPA, we used siRNA to target p300 and PCAF. However, our data do not indicate involvement of PCAF. We believe that siRNA is a more specific tool than pharmacologic inhibitors, which often have broad span of effects, and this could be the reason for the discrepancy. Regarding p300, our data imply a critical role of this coactivator for the maintenance of basal t-PA expression but no involvement in the VPA-response. Certainly, the magnitude of the t-PA induction was reduced in our p300 experiments, but we state the independence of p300 in VPA-mediated t-PA induction based on almost identical VPA-responses when calculated relative to respective unstimulated control.

## 5. Conclusion

We examined the mechanism of how the histone deacetylase (HDAC) inhibitor valproic acid (VPA) induces the endothelial fibrinolytic enzyme t-PA. In summary, we show that VPA-mediated induction of t-PA is dependent on the proximal GC boxes in the t-PA promoter and possibly involves interaction with (acetylated) transcription factors Sp2, Sp4, and KLF5.

## Supplementary Material

The supplementary information includes three tables (A–C) showing: (a) Gene regulation data of transcription factors from the Sp/KLF family in response to VPA. (b) The Sp/KLF transcription factors that were selected for siRNA knock down experiments and the re-agents that were used. (c) The reagents that were used for siRNA knock down experiments of p300 and PCAF.
