# Supplementary material for: Dependence of Proximal GC Boxes and Binding Transcription Factors in the Regulation of Basal and Valproic Acid-Induced Expression of t-PA
Source: Int J Vasc Med. 2016 Feb 7;2016:7928681. doi: 10.1155/2016/7928681 (PMC4761389; doi:10.1155/2016/7928681)
Supplement: Supplementary file 1 — The supplementary information includes three tables (A–C) showing: (a) Gene regulation data of transcription factors from the Sp/KLF family in response to VPA. (b) The Sp/KLF transcription factors that were selected for siRNA knock down experiments and the re-agents that were used. (c) The reagents that were used for siRNA knock down experiments of p300 and PCAF. [file 7928681.f1.pdf]

Article: Dependence of Proximal GC-boxes and Binding Transcription Factors in the Regulation of Basal and Valproic Acid-Induced Expression of t-PA

Journal: International Journal of Vascular Medicine

Erik Ulfhammer[1], Pia Larsson[1], Mia Magnusson[1], Lena Karlsson[1], Niklas Bergh[1] and Sverker Jern[1]

[1] The Wallenberg Laboratory for Cardiovascular Research, Institute of Medicine,

The Sahlgrenska Academy, University of Gothenburg, Gothenburg, Sweden.

Correspondence to: Sverker Jern, M.D., E-mail: sverker.jern@gu.se

Online Resource 1. Regulation of Sp and KLF transcription factors by VPA in HUVEC and presentation of selected proteins for siRNA studies

A.

Gene	Fold change
sp1	0.96
sp2	0.72
sp3	1.04
sp4	1.26
sp5	1.05
sp6	1.20
sp7	0.97
sp8	0.96
sp9	1.02
KLF1	1.06
KLF2	0.98
KLF3	1.10
KLF4	1.31
KLF5	1.33
KLF6	0.98
KLF7	1.03
KLF8	1.03
KLF9	1.30
KLF10	0.69
KLF11	0.91
KLF12	1.08
KLF13	1.23
KLF14	1.00
KLF15	0.99
KLF16	1.00
KLF17	0.93

B.

Gene	Selection criteria	siRNA	Gene Expression Assay
sp1	Endothelial described, t-PA context	L-026959-00-0005	Hs00916521_m1
sp2	Regulated	L-015955-00-0005	Hs00370726_m1
sp3	Endothelial described, t-PA context	L-023096-00-0005	Hs01595811_m1
sp4	Regulated	L-006562-00-0005	Hs00162095_m1
KLF2	Endothelial described	L-006928-00-0005	Hs00360439_g1
KLF4	Regulated, Endothelial described	L-005089-00-0005	Hs00358836_m1
KLF5	Regulated	L-013571-00-0005	Hs00156145_m1
KLF6	Endothelial described	L-021441-00-0005	Hs00810569_m1
KLF9	Regulated	L-011223-00-0005	Hs00230918_m1
KLF10	Regulated	L-006566-00-0005	Hs00921811_m1
KLF13	Regulated	L-006994-00-0005	Hs00429818_m1
KLF15	Endothelial described	L-006975-00-0005	Hs00362736_m1

C.

Gene	siRNA	Gene Expression Assay
p300	L-003486-00-0005	Hs_EP300_1_SG
PCAF	L-005055-00-0005	Hs00187332_m1

A. HUVECs (n=4) were stimulated with 4 mM VPA for 24 h and analyzed by microarray for alteration in gene expression. The table shows expression of all characterized Sp and KLF transcription factors [1, 2] relative to untreated control cells. Data from these experiments were recently presented elsewhere [3] and the total set of raw data are available at http://www.ncbi.nlm.nih.gov/geo/query/acc.cgi?token=dnglzumweoisshk&acc=GSE23909.

B. Selected Sp and KLF factors for siRNA knock down studies. The selection criteria was either significantly regulated ($p<0.001$) by VPA in microarray experiments [3] or previously described in an endothelial gene regulation context; Sp1 [4] , Sp3 [5] , KLF2 [6] , KLF4 [7] , KLF6 [8], KLF15 [9]. The table also shows product numbers of used siRNA (Dharmacon) and gene expression assays (Applied Biosystems). C. Products used for siRNA (Dharmacon) depletion of p300 and PCAF as well as gene expression assays for p300 (Qiagen) and PCAF (Applied Biosystems).

References:

1. McConnell BB, Yang VW (2010) Mammalian Kruppel-like factors in health and diseases. Physiol Rev 90(4):1337-1381.
2. Wierstra I (2008) Sp1: emerging roles--beyond constitutive activation of TATA-less housekeeping genes. Biochem Biophys Res Commun 372(1):1-13.
3. Larsson P, Ulfhammer E, Magnusson M, Bergh N, Lunke S, El-Osta A, Medcalf RL, Svensson PA, Karlsson L, Jern S (2012) Role of histone acetylation in the stimulatory effect of valproic acid on vascular endothelial tissue-type plasminogen activator expression. PLoS One 7(2):e31573.
4. Arts J, Herr I, Lansink M, Angel P, Kooistra T (1997) Cell-type specific DNA-protein interactions at the tissue-type plasminogen activator promoter in human endothelial and HeLa cells in vivo and in vitro. Nucleic Acids Res 25(2):311-317.

5. Merchiers P, Bulens F, De Vriese A, Collen D, Belayew A (1999) Involvement of Sp1 in basal and retinoic acid induced transcription of the human tissue-type plasminogen activator gene. FEBS Lett 456(1):149-154.
6. Kuo CT, Veselits ML, Barton KP, Lu MM, Clendenin C, Leiden JM (1997) The LKLF transcription factor is required for normal tunica media formation and blood vessel stabilization during murine embryogenesis. Genes Dev 11(22):2996-3006.
7. Yet SF, McA'Nulty MM, Folta SC, Yen HW, Yoshizumi M, Hsieh CM, Layne MD, Chin MT, Wang H, Perrella MA, Jain MK, Lee ME (1998) Human EZF, a Kruppel-like zinc finger protein, is expressed in vascular endothelial cells and contains transcriptional activation and repression domains. J Biol Chem 273(2):1026-1031.
8. Kojima S, Hayashi S, Shimokado K, Suzuki Y, Shimada J, Crippa MP, Friedman SL (2000) Transcriptional activation of urokinase by the Kruppel-like factor Zf9/COPEB activates latent TGF-beta1 in vascular endothelial cells. Blood 95(4):1309-1316.
9. Helbing T, Volkmar F, Goebel U, Heinke J, Diehl P, Pahl HL, Bode C, Patterson C, Moser M (2010) Kruppel-like factor 15 regulates BMPER in endothelial cells. Cardiovasc Res 85(3):551-559.
